# Genetic Health Education in Adolescents with Congenital Heart Disease: A Patient, Parent, and Clinician Perspective

**DOI:** 10.1007/s00246-025-04010-4

**Published:** 2025-09-16

**Authors:** Bridget R. O’Malley, Janine Smith, Gary F. Sholler, Julian Ayer, Gillian M. Blue

**Affiliations:** 1 The Heart Centre for Children, Sydney Children’s Hospitals Network, Sydney, NSW 2145 Australia; 2 The Faculty of Medicine and Health, The University of Sydney, Sydney, Australia; 3 Department of Clinical Genetics, The Children’s Hospital at Westmead, Sydney, Australia

**Keywords:** Congenital heart disease, Adolescents, Education, Genetics, Recurrence

## Abstract

Improvements in outcomes for congenital heart disease (CHD) have resulted in a growing adolescent/adult CHD population. Subsequently, education on CHD causes and inheritance has been identified as important. This study aimed to determine population-specific understanding and preferences of CHD causes and inheritance education. CHD adolescents aged 13–18 years, their parent, and clinicians involved in the care of patients with CHD completed a purpose-designed, online survey outlining their current understanding of CHD genetics and inheritance and their preferences for education content and delivery. Approximately 50% of participants were unsure if CHD was caused by genetic factors, and 25% believed CHD was not genetic. Many participants (63% (19/30) adolescents, 81% (26/32) parents) wanted to know more about CHD causes, with most considering this important. Over 60% of participants indicated that CHD causes had not been discussed with them, despite 74% of clinicians (17/23) indicating they discuss this. Information on genetic causes of CHD and recurrence risks, delivered in-person with parents present, was the preferred option for the content and delivery of this information. Most clinicians indicated genetics education should be introduced between 12 and 14 years, whereas adolescents and parents preferred this to occur between 14 and 16 years. Participants want to know more about CHD causes and inheritance, in an outpatient setting with parents in attendance. The findings of this study support the development of a specialized genetics education program about CHD causes and inheritance for adolescents with CHD. Further, it provides important insight into the content, timing, and delivery of this information.

**Supplementary Information:**

The online version contains supplementary material available at 10.1007/s00246-025-04010-4.

## Introduction

Congenital Heart Disease (CHD) is the most prevalent congenital abnormality, affecting approximately 9.4/1000 babies born globally [1, 2]. CHD is a clinically heterogeneous condition that encompasses various structural lesions affecting the heart, heart valves, or central blood vessels [1]. Improved outcomes for CHD patients, with 97% of affected children surviving to adulthood, have resulted in an expanding adolescent and adult CHD population, with an estimated annual increase of ~ 5% [3]. Subsequently, addressing outcomes, such as long-term health and quality of life, is increasingly relevant for this population [4].

The etiology of CHD is complex and, in most cases (~ 80%), is thought to be multifactorial, with both genetic and environmental (maternal–fetal-placental) factors contributing to disease development. Approximately 20–30% of CHD cases are attributed to known causes, including genetic and environmental exposures [5, 6]. Recurrence rates in CHD can vary considerably depending on whether the condition is familial, if it occurs in isolation or with other non-cardiac health issues [5]. As such, having a better understanding of the causes of CHD and associated recurrence risks is important for adolescents and young adults with CHD as this has important consequences for family planning. While genetics care in CHD patients is rapidly evolving with increased diagnostic yields in certain CHD patient groups [7], most adolescents with CHD in Australia will not have been exposed to clinical genetics care or consultations with genetic health professionals.

The shift in disease outlook as a result of improved long-term outcomes for CHD has emphasized the importance of effective transition processes from pediatric to adult cardiac care, including education and psychosocial support [8–11]. Adults with CHD lack sufficient understanding of the genetic causes and recurrence risks of CHD [12, 13]. The value of including this information in the transition process has been recognized, with established recommendations for clinical practice [9, 11, 14–16]. Specifically, studies have shown that adolescents with CHD and their parents had little understanding of the genetic causes of CHD, despite a high level of interest in learning more about this [17]. To the best of our knowledge, there is no specialized genetics education program for adolescents with CHD to provide accurate information about the genetics and recurrence of CHD.

This study aimed to determine population-specific understanding and preferences of CHD causes and inheritance education in Australian adolescents with CHD, their parents, and clinicians involved in CHD care, to inform the development of a specialized genetics education program. This work builds on the literature [17], through the inclusion of a more multicultural study cohort representative of the Australian population, greater representation of CHD lesions across the severity spectrum and, importantly, the inclusion of clinicians’ perspectives on this topic.

## Methods

### Participant Recruitment

This study was approved by the Sydney Children’s Hospital Network (SCHN) Human Research Ethics Committee (2022/ETH01064). Over a 12-month period, adolescents with CHD aged 13–18 years and their parent/guardian (participants) were prospectively recruited via attendance at routine cardiology and transition outpatient clinics at the Sydney Children’s Hospital Network (SCHN). Parent/guardian participation was limited to one parent/guardian. Once consented, participants completed a purpose-designed, online survey outlining their current understanding of CHD genetics and inheritance, and their preferences for education content and delivery. Participants were assigned a unique ID number, with parent and adolescent dyads linked via a family ID. Parents/guardians of adolescents with a diagnosed syndrome with associated neurodevelopmental delay were approached and were able to determine their child’s capacity to participate. Non-English-speaking participants requiring an interpreter were approached if an interpreter was present. Participants’ electronic medical records were used to confirm cardiac and genetic diagnoses, and any engagement with clinical genetics services at SCHN.

### Survey Development, Dissemination, and Data Collection

Purpose-designed surveys were developed in consultation with pediatric cardiologists and clinical geneticists, with complementary surveys developed for adolescents and their parent/guardian. This ensured age-appropriate content was contained in the respective surveys, which comprised of the following four domains:

A. Information about you (nine items)
  Included items to ascertain basic participant demographic data including gender, age, education, and rural status.
B. About your heart condition (nine items)
  Participants were asked a series of questions about their heart condition including the name of their heart condition, if anyone had discussed possible causes with them, if anyone else in their family had a similar heart condition, and if they had seen a geneticist for their heart condition. Additionally, a series of statements asked participants to rate the impact their/their child’s heart condition would have on various aspects of their/their child’s life from “very likely” to “very unlikely.”
C. Your understanding of heart disease causes (six items)
  Adolescents and parents were asked to respond to the statement “I believe I have a heart condition because…” as a free text response. Multiple choice questions then assessed participants understanding of CHD causes, including genetic and environmental causes, and recurrence risks. Participant interest in learning more about CHD causes and the importance of this was also assessed.
D. Your preferences for a genetics education program (six items)
  This section comprised participant preferences and interest in a genetics education program, including topic preferences, the age this information should be introduced, family member and clinician involvement, and preferred information delivery and settings. Participants could also indicate their interest to be contacted should a program become available in the future.

The link to the online survey, hosted on REDCap, was sent to participants nominated email address and took ~ 5–10 min to complete on their own device.

Clinicians involved in the care of CHD patients at SCHN and nationally via the Cardiac Society of Australia and New Zealand’s (CSANZ) Paediatric and Congenital Council, completed a survey to identify current practices and preferences for the delivery of this information. All clinicians were contacted via email and survey completion was considered implied consent. Some participating clinicians may have been direct care providers to participating adolescents; however, due to the anonymous nature of the clinician survey this cannot be confirmed.

### Data and Statistical Analysis

Participant and clinician responses were analyzed initially as three distinct groups and subsequently as paired adolescent–parent dyads. Descriptive statistics were used to describe demographic and clinical characteristics, and appropriate descriptive and statistical analyses were applied. Chi-squared analyses were used to evaluate correlations between participant demographics and clinical characteristics and items assessing their understanding of genetic causes of CHD and recurrence risks. Additional analyses compared responses between adolescents, parents, and clinicians. All statistical analyses were completed using SPSS version 25.

## Results

### Adolescent and Parent Analysis

**Fig. 1:**
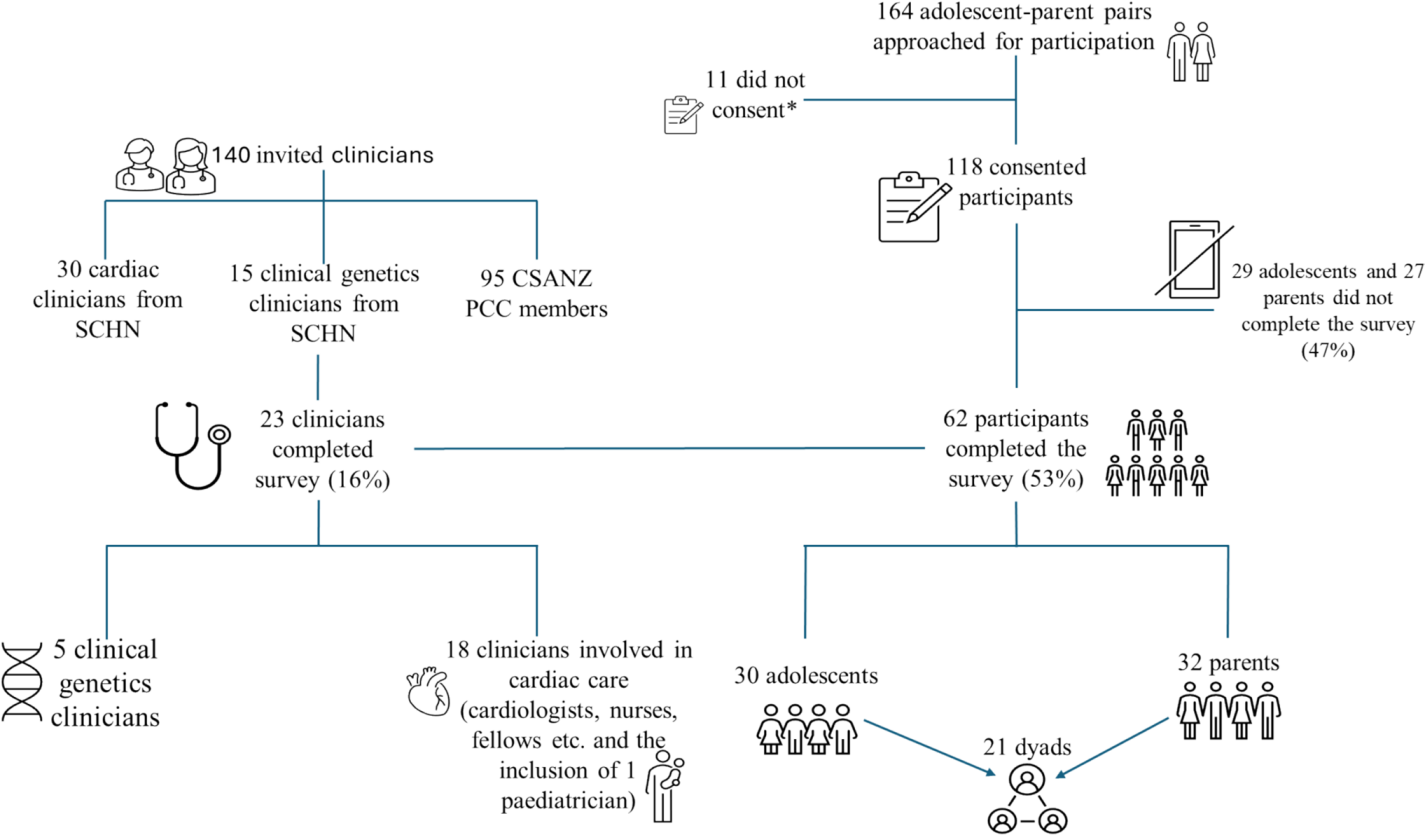
Study recruitment and participant response rate. *Reasons for not consenting included being under 18 years of age with no parent to co-consent and not wanting to participate

Over a 12-month period, 118 participants were consented to the study and 62 participants completed the survey (30 adolescents and 32 parents), resulting in a response rate of 53% (Fig. 1). Approached adolescents represent approximately 79% of eligible patients attending cardiology clinic appointments during the recruitment period. Participant demographic characteristics are shown in Table 1. 53% of adolescents identified as Caucasian, followed by Arabic (17%) and South Asian (10%), with similar representation among parent/guardian participants (Table 1). The range of CHD severity was well represented with 46% (14/30) adolescents exhibiting complex CHD, and the remainder simple or moderate disease [18]. 7% (2/30) of adolescents and 12% (4/32) parents reported a family history of CHD (Table 1).

**Table 1:** Participant demographic characteristics

Demographic variable	Adolescent participants (*n*)	Parent/guardian participants (*n*)
*Gender*		
Female	13 (43%)	25 (78%)
Male	17 (57%)	7, (22%)
Other	0	0
*Age*		
13–14	10	0
15–16	13	0
17–18	7	0
30–39	0	4
40–49	0	18
50–60	0	5
Unspecified	0	5
*Ancestry/ethnicity (participants were able to select up to two options)*		
Caucasian	16 (53%)	20 (63%)
Aboriginal/Torres Strait Islander	1 (3%)	0 (0%)
Arabic	5 (17%)	4 (13%)
South Asian	3 (10%)	3 (9%)
East Asian	0 (0%)	0 (0%)
South-East Asian	2 (7%)	2 (6%)
Pacific Islander	1 (3%)	0 (0%)
Mixed ethnicity	2 (7%)	3 (9%)
*Geographic*		
Metro	23 (77%)	22 (69%)
Rural	6 (20%)	9 (28%)
Did not respond	1 (3%)	1 (3%)
*Household type*		
Both parents living in the same household	23 (76.7%)	25 (78.1%)
Sole parent family after divorce/separation	5 (16.7%)	4 (12.5%)
Sole parent family, other parent not involved or deceased	0	1 (3.1%)
Grandparents are sole carers	0	0
Blended family	2 (6.7%)	1, 3.1%
Foster family	0	0
Other	0	1, 3.1%
*Education*	*Type of school attended:*	*Highest level of education:*
	Private school (including catholic and independent schools): 9 (30%)	Primary school: 0
	Public school: 21 (70%)	High school: 8 (25%)
	*Currently in:*	TAFE or college certificate or diploma: 9 (28%)
	Year 7: 3 (10%)	Trade/apprenticeship: 0
	Year 8: 4 (13.3%)	University graduate: 10 (31%)
	Year 9: 4 (13.3%)	Postgraduate study: 5 (16%)
	Year 10: 6 (20%)	Other: 0
	Year 11: 3 (10%)	
	Year 12: 7 (23.3%)	
	Completed year 12: 3 (10%)	
	Left school prior to year 12: 0	
*Have you learnt about genetics at school (e.g., genes, chromosomes, Mendelian inheritance or punnet squares)?*	Yes: 14 (47%)	
	No: 16 (53%)	
*Oral health rating*		
Excellent	2 (7%)	5 (16%)
Very good	14 (47%)	12 (39%)
Good	10 (33%)	6 (19%)
Fair	4 (13%)	7 (23%)
Poor	0	1 (3%)
*CHD type (broad categories)*		
ASD	2 (7%)	2 (6%)
VSD	6 (20%)	6 (19%)
AVSD and associated variants	1 (3%)	3 (9%)
Malformation of outflow tract	10 (33%)	9 (28%)
Obstructive	3 (10%)	4 (13%)
Functional single ventricle	4 (13%)	5 (16%)
Heterotaxy	0	0
Other	4 (13%)	3 (9%)
*CHD lesion correctly identified*		
Yes	24 (80%)	29 (91%)
No	6 (20%)	3 (9%)
*Family history of CHD*		
Yes	2 (7%)	4 (12%)
No	28 (93%)	28 (88%)
*Genetic diagnosis explaining CHD *		
Yes	3 (10%)	
No	27 (90%)	
*Genetics consult (electronic medical record)*		
Yes	7 (23%)	
No	23 (77%)	
*Genetics consult (self reported)*		
Yes	2 (7%)	6 (19%)
Yes—not in relation to the heart condition	2 (7%)	2 (6%)
No	17 (57%)	19 (59%)
Unsure	9 (30%)	5 (16%)

**Fig. 2:**
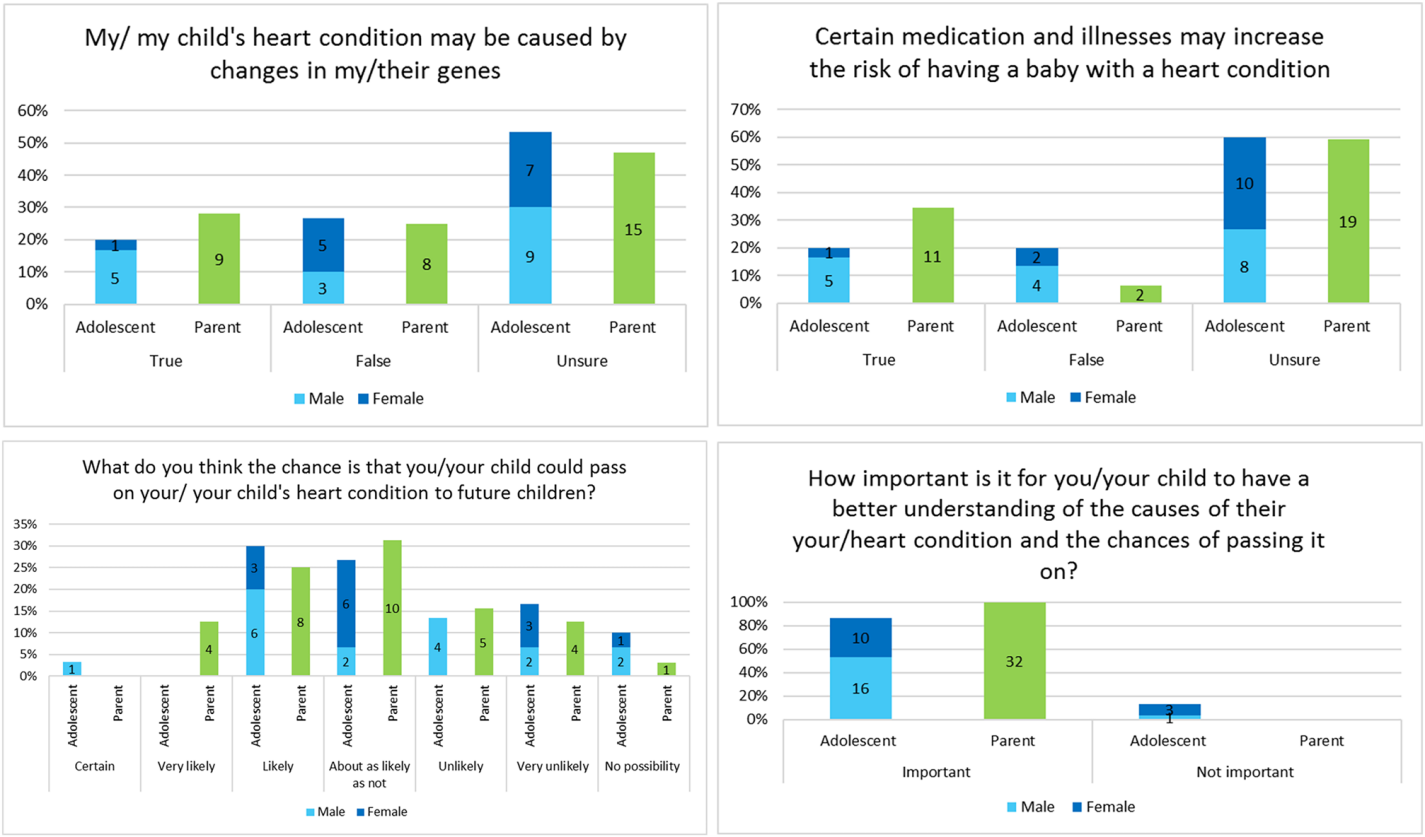
Participant understanding and importance of CHD causes and inheritance. *n* = shown in center of each bar

Adolescent and parent participants were largely unsure if CHD was caused by genetic factors with 53% (16/30) of adolescents and 47% (15/32) of parents indicating this. Approximately, a quarter of adolescent (26%, 8/30) and parent (25%, 8/32) participants believed CHD was not genetic (Fig. 2). There was no significant difference between adolescents and parents in their understanding of genetic causes of CHD (*p* = 0.755, Fig. 2). 48% (14/30) of adolescents stated they had learnt about genetics at school (Table 1) and this was not significantly associated with having a better understanding of CHD genetic causes (*p* = 0.378) or recurrence (*p* = 0.529). Despite 72% (13/18) of clinicians indicating they discuss CHD causes, over 60% of participants (63%, 19/30 of adolescents and 66%, 21/32 of parents) indicated this had not been discussed with them. More male adolescents reported that they had engaged in these discussions (53%, 9/17), compared to their female counterparts (15%, 2/13, *p* = 0.057) and that this was mostly with their parents and/or cardiologist. Parent recollections of their experience of these discussions are highlighted by the free text responses:

"We were told left heart defects are not genetic and no known causes are known."

"He was just born with it. No reason for it. Advised by the doctor at 5 days old."

In terms of recurrence risks, approximately 33% (10/30) of adolescents and 38% (12/32) of parents indicated that they/their child could pass on their heart condition to their children (Fig. 2). Interestingly, one adolescent indicated it was ‘certain’ that they would pass on CHD to their children; and three adolescents and one parent thought there was no chance of this.

**Fig. 3:**
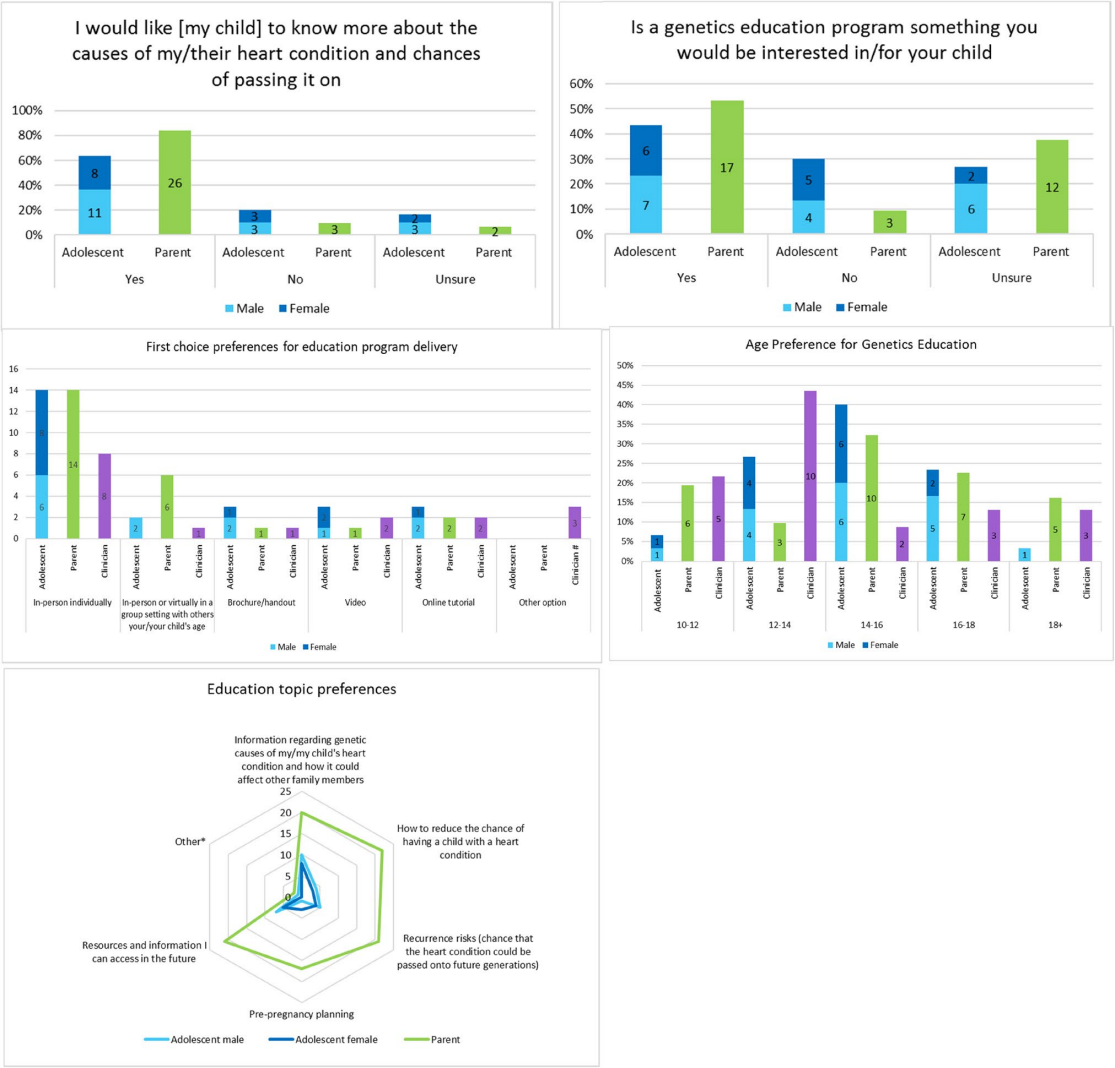
Participant interest and preferences for CHD genetics education. #A multistage approach, with written information provided followed by tailored counselling. *Impact on ability to exercise and weightlifting, contact at the Children’s Hospital once their child leaves at 18 years and one parent did not provide a response

Both adolescents and parents highly valued education about CHD causes and recurrence, with 100% (32/32) of parents and 87% (26/30) of adolescents considering this important (Fig. 2). In line with this, 81% (26/32) of parents and 63% (19/30) of adolescents wanted to learn more, with similar interest in being contacted for participation in a specialized program, should one become available (60% (18/30) adolescents and 77% (24/31) parents; Fig. 3). Parents were eager for their child to learn about all suggested topics and were most interested in risk reduction, recurrence, and resources to access in the future. In comparison, adolescents wanted to learn about CHD causes, effect on family members, and resources they could access in the future (Fig. 3). Most participants indicated that genetics information should be introduced at age 14–16 years (Fig. 3) and most adolescents (60%, 18/30) indicated that they wanted their parents present during discussions.

### Dyad Analysis

Dyad analysis assessed parent–adolescent responses among the 21 dyad pairs across all survey items. There were no significant differences in responses between parent and adolescent dyads across any of the survey items.

### Clinician Analysis

23/140 contacted clinicians completed the survey, resulting in a response rate of 16% (Fig. 1). Responses received included those from local clinicians and clinicians practicing around Australia. The cohort was broken down by specialty with those who primarily provide cardiac care (e.g., cardiologist), including 1 pediatrician and 3 cardiac specialty nurses, referred to as ‘cardiac clinicians’ (*n* = 18) and those providing primarily genetics-related care as ‘genetic clinicians’ (*n* = 5) (Supplementary Fig. 1). Most clinicians had over 15 years of experience (Supplementary Fig. 1) and provided care to pediatric, or both pediatric and adult CHD patients.

Most cardiac clinicians (61%, 11/18) were confident in their CHD genetics knowledge and were comfortable discussing this information (72%, 13/18). When asked to elaborate on the topics they discuss, cardiac clinicians indicated that they ‘always’ and/or ‘often’ discuss ‘CHD causes’ (10/12), ‘CHD recurrence’ (10/14), ‘Genetic testing availability’ (9/13), and ‘Options for referrals to genetic services’ (12/17) (Supplementary Fig. 2).

All clinicians (23/23) thought that it was important for patients and their families to understand CHD causes and inheritance, with all cardiac clinicians (18/18) and most (4/5, 80%) clinical genetics clinicians indicating that a specialized genetics education program would be beneficial for their patients. Nearly half of all clinicians thought genetics education should be introduced at ages 12–14 years (43%, 10/23) (Fig. 3). Cardiologists were identified as being the most appropriate person to introduce CHD genetics discussions (65%, 15/23) and a genetic counselor (61%, 14/23) as the ideal person to discuss detailed information with patients. Both cardiac and genetic clinicians identified the ideal setting for the delivery of CHD genetics information as individually, in-person (Fig. 3).

## Discussion

To date, there is limited data on CHD adolescents’ understanding of CHD genetics and inheritance. This study aimed to investigate current understanding of CHD genetics and recurrence in adolescents with CHD and their parents, preferred delivery modes, and content of a specialized genetics education program in our unique Australian population. Expanding on work by Crawford et al. [17], with the inclusion of the clinician perspective, greater representation across disease severity and cultural diversity, this study aimed to inform the development of a specialized CHD genetics education program, to be implemented into routine cardiac care of adolescents with CHD.

The importance of effective education for adolescents with CHD transitioning from pediatric to adult cardiac care is well described [8–11]. Education about recurrence risks was identified as an important area for improvement [9, 11, 12, 14, 15], however, little evidence exists about best practices for the content and delivery of this information. This study identified that both adolescents and parents highly valued education about CHD causes and recurrence risks, with most indicating they wanted to learn more about this. Participants also thought having a good understanding of CHD causes and recurrence risks was important.

### Current Understanding and Experiences

Most participants had a good understanding of their CHD, contrasting previous reports [19]. Participant recounts of receiving information on CHD causes in our cohort were low, albeit consistent with the literature [12, 16], highlighting the importance of ongoing and repeated discussions to reinforce the topic. Further, parents indicated that these discussions typically occurred following a clinical suspicion of a syndromic diagnosis, however, for most patients with isolated CHD, parents could only recall limited information about the possible causes being provided. Importantly, a quarter of study participants did not think that CHD may be caused by genetic factors, with most indicating they were ‘unsure’ and only about a third of participants thought that CHD could be passed onto future generations, similar to previous reports [17]. This highlights the need for improved genetics education in this patient group, specifically on the causes and inheritance/recurrence of CHD. While recurrence risks are generally low for isolated CHD, they can vary considerably between specific CHD types and clinical presentations and in some cases can be as high as other monogenic diseases. It is therefore important that patients are aware of these increased risks and whether they relate to their specific clinical presentation as they enter reproductive age.

Clinicians were confident in their understanding of CHD genetics and their ability to relay basic information to their patients about CHD causes and recurrence, with most clinicians (72%) indicating that they provide this information to most or all their patients and take as long as needed to explain this information.

### Program Delivery Preferences

The ideal setting to deliver genetics education was individually in-person, with parents present, in conjunction with a routine cardiology appointment at 14–16 years of age. This is in line with reports in the literature where participants preferred in-person education session, at ~ 16 years of age and with parents and clinicians present [9, 17]. Adolescent and parental preferences for an education program were largely similar. Adolescents highly favored in-person delivery of education, and surprisingly, showed little interest in e-based learning options, however, this is reflective of current literature [17]. Our study expanded on previous reports in terms of the content of the genetic education with participants being able to indicate their preferences on topics covered. Adolescents were mostly interested in CHD causes, whereas parents’ preferences were high across all topics, possibly reflecting a greater interest in any information related to this topic having already had a child with CHD.

Clinicians were supportive of a specialized genetics education program and mirrored participant preferences in terms of the setting and mode of delivery. They also indicated cardiologists as the most appropriate person to engage in initial discussions about CHD causes and inheritance and for a genetics clinician to provide more detailed information on this. Interestingly, clinicians indicated that the education session should be delivered earlier between the ages of 12 and 14 years, suggestive of the relevance at this life stage and an increased confidence in their patients’ abilities to absorb the information at a younger age.

The findings of this study support the development of a genetics education program for adolescents with CHD, as follows:

- To deliver a voluntary individual, in-person genetics education program to patients with CHD, built into existing transition clinics and/or cardiac appointments, preferably between the ages of 14 and16 years
- To have the option of parents/additional family members/support person in attendance at the genetics education program
- To cover topics including: (1) the causes of CHD, (2) inheritance and recurrence risks of CHD, (3) risk reduction in CHD, (4) available resources and information, and (5) a brief overview of pre-pregnancy planning

We acknowledge that these preferences represent those of the study participants and may not consider practical aspects, including resourcing and scalability beyond specialist centers/services. As such, this study also recommends:

- Ongoing conversations, relevant to life stage, between cardiologists, parents, and young people with CHD about CHD causes and inheritance to reinforce this information over time to ensure retention and relevancy.
- Earlier preliminary discussions on CHD causes and inheritance introduced to adolescents and parents by cardiologists from the ages of 12–14 years to raise awareness of the topic.
- Exploring other avenues, such as e-based options, as alternatives in cases where resources are mismatched to demand and to address feasibility and scalability.
- Opportunities and resources adolescents can access in future, particularly for topics related to recurrence and pre-pregnancy planning.

Future work should focus on the development, implementation, and evaluation of a specialized genetics education program as part of routine cardiac care for adolescents with CHD. Additionally, surveys on preferences for genetic information in other age groups, including at time of diagnosis and during follow-up in childhood, to enable appropriate communication of this information throughout a CHD patient’s life, are important. While this work and that of others support an individual, in-person genetics education program, future work should focus on the practicalities, including the allocation and funding of the many resources required, to facilitate this. Acknowledging ideal participant preferences, other avenues, such as e-based options, may need to be explored as alternatives in cases where resources are mismatched to demand. Finally, further investigation into the effectiveness of genetics education for patients with CHD, particularly in the long term and how this information may inform and promote improved healthcare outcomes, also in terms of family planning, for this patient group will be needed.

### Limitations

There are several limitations to consider in this study. As with most surveys, there will be participation bias, with those study participants and clinicians more interested and/or familiar with the survey topic, more likely to participate, than those that are not. The low survey response rate by clinicians limits the applicability of clinician practices and preferences and the development of the educational program would benefit from broader clinician input. Further, as study participants were recruited during their cardiology appointments, this may have influenced their responses, particularly their understanding of their heart defect. While participants with a broad range of cardiac lesions were recruited to the study, including some with less severe/complex disease, they were recruited from outpatient cardiac clinics typically requiring ongoing care, such that those patients no longer engaging in the service, being excluded. Similarly, while study participants represent a culturally diverse population, representative of the largest tertiary pediatric cardiac center in New South Wales, the findings are limited to a single institution. Finally, the views and experiences of parent participants, most likely reflect experiences and information received at the time of their child’s surgery which may be > 17 years ago and therefore, may not reflect current practices and experiences.

## Conclusion

In summary, adolescents with CHD require an effective transition process, equipping them with the required skills and knowledge to navigate this shift in medical autonomy. Having a sound understanding of the genetic causes of CHD and the associated recurrence risks is key information as they begin to contemplate this. While study limitations including participation and recall bias need to be acknowledged, the findings of this study support the development of a specialized genetics education program for all adolescents with CHD and provide important insight into the content, timing, and delivery of this information.

## Supplementary Information

Below is the link to the electronic supplementary material.

Supplementary file1 (DOCX 399 KB)

## Data Availability

No datasets were generated or analysed during the current study.
