# Correction: Genetic Health Education in Adolescents with Congenital Heart Disease: A Patient, Parent, and Clinician Perspective

**DOI:** 10.1007/s00246-025-04050-w

**Published:** 2025-10-31

**Authors:** Bridget R. O’Malley, Janine Smith, Gary F. Sholler, Julian Ayer, Gillian M. Blue

**Affiliations:** 1 The Heart Centre for Children, Sydney Children’s Hospitals Network, Sydney, NSW 2145 Australia; 2 The Faculty of Medicine and Health, The University of Sydney, Sydney, Australia; 3 Department of Clinical Genetics, The Children’s Hospital at Westmead, Sydney, Australia

**Correction to: Pediatric Cardiology** 10.1007/s00246-025-04010-4.

In this article Fig. 2 appeared incorrectly and have now been corrected in the original publication. For completeness and transparency, the old incorrect version and corrected version are displayed below.

Incorrect Figure
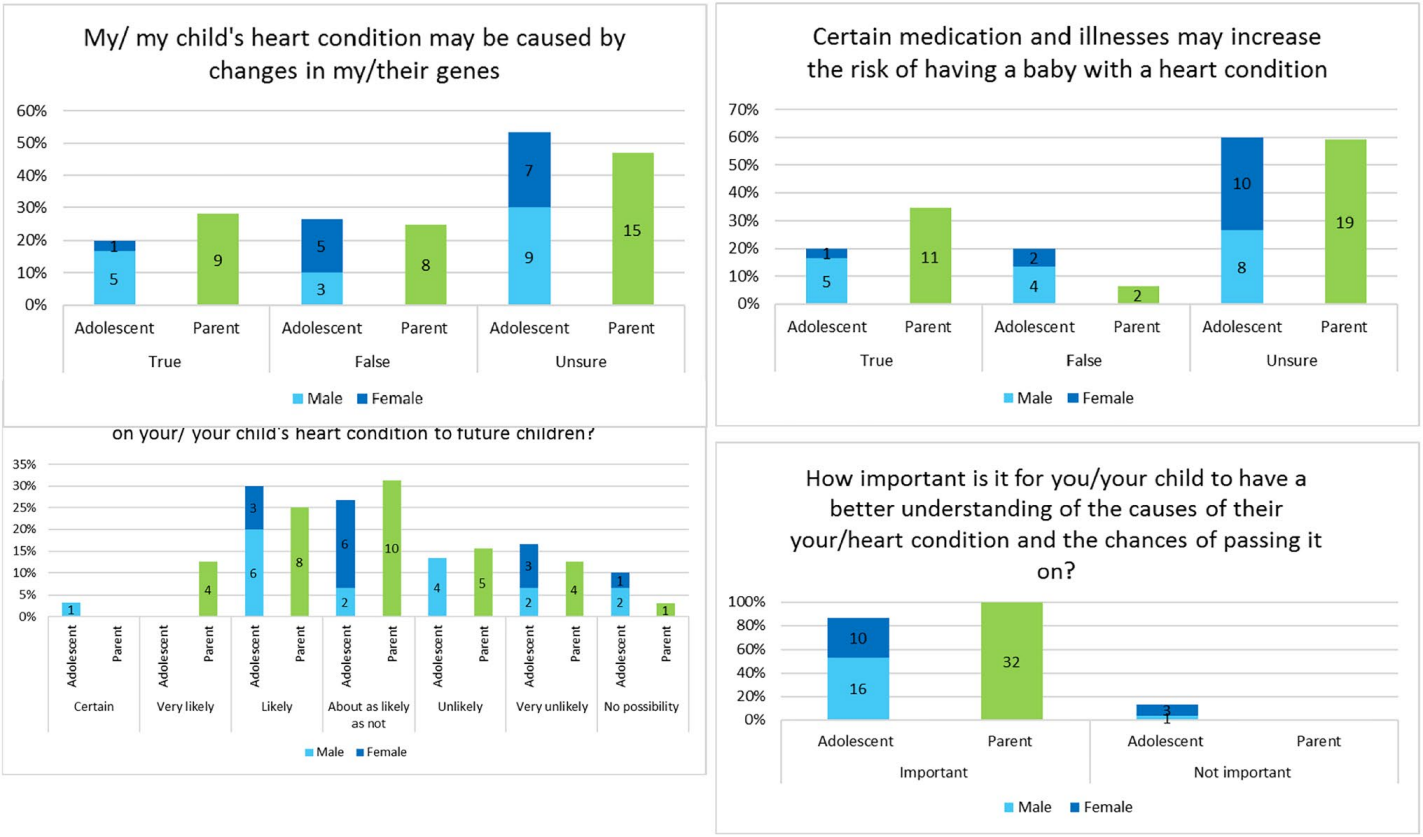


Corrected Figure

**Fig. 2:**
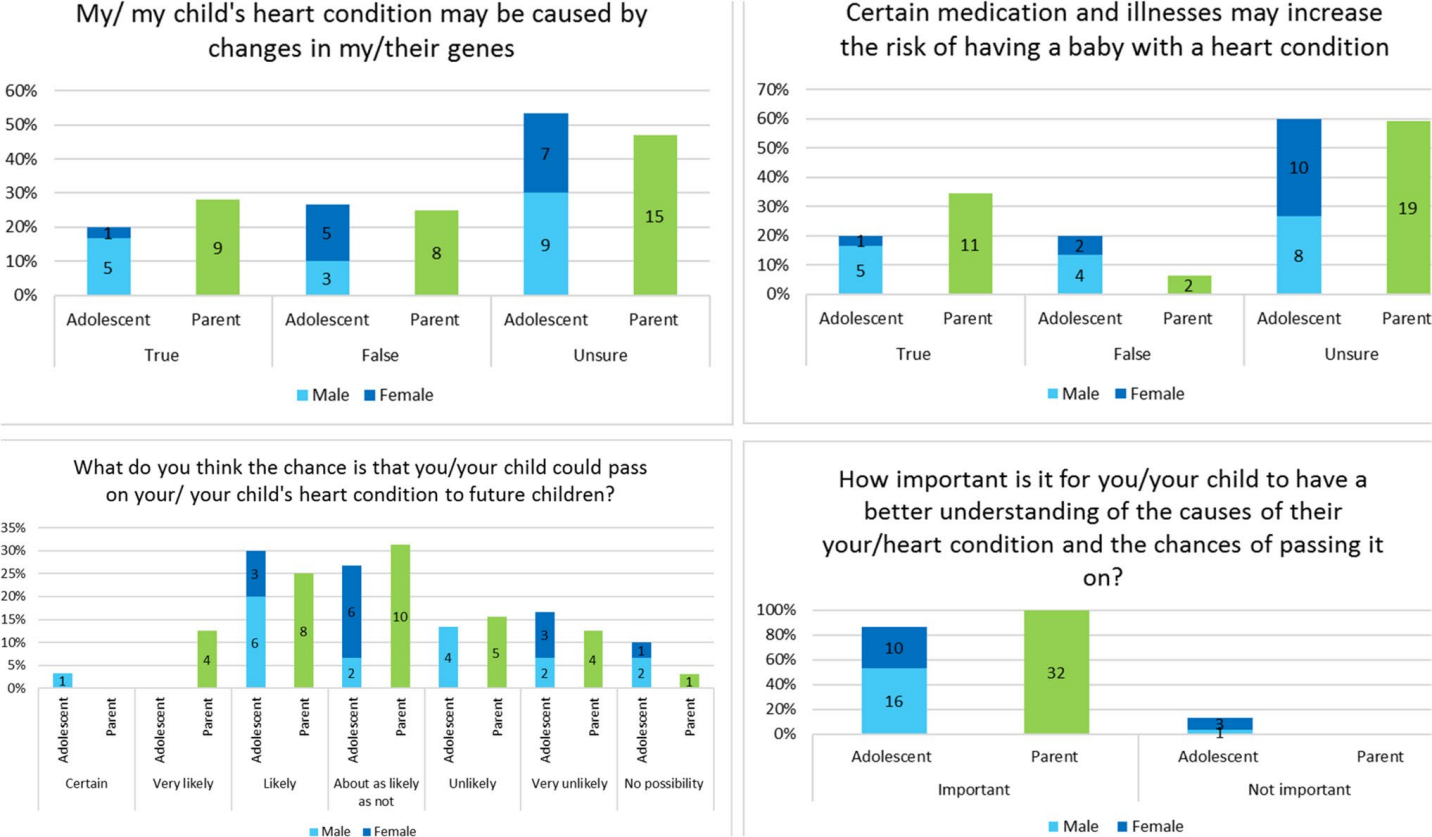
Participant understanding and importance of CHD causes and inheritance. *n* = shown in center of each bar

The original article has been corrected.

