# Supplementary material for: Genetic Health Education in Adolescents with Congenital Heart Disease: A Patient, Parent, and Clinician Perspective
Source: Pediatr Cardiol. 2025 Sep 16;47(5):2182–91. doi: 10.1007/s00246-025-04010-4 (PMC13144196; doi:10.1007/s00246-025-04010-4)
Supplement: Supplementary file 1 — Supplementary file1 (DOCX 399 KB) [file 246_2025_4010_MOESM1_ESM.docx]

Supplementary Materials

**Supplementary Figures**


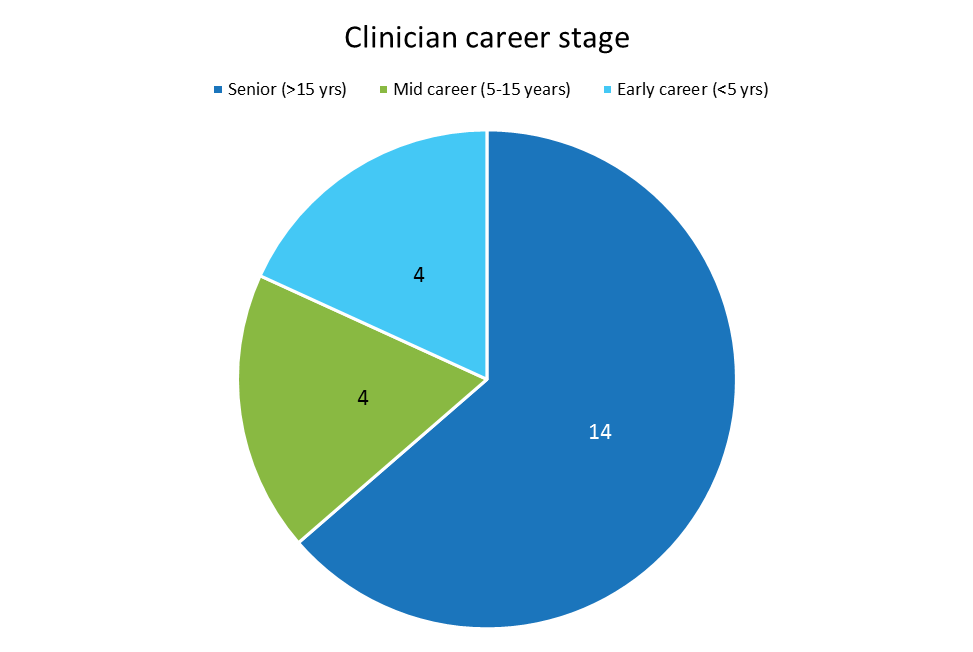


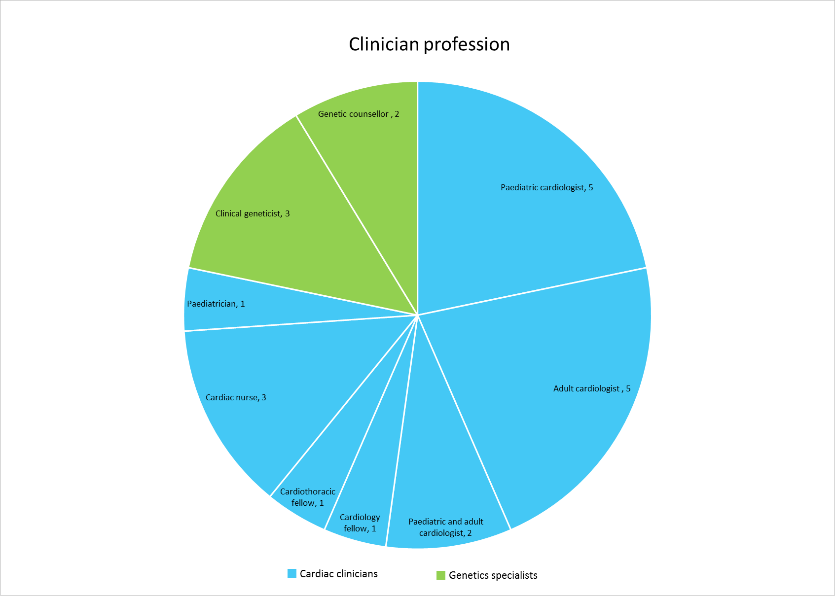


Supplementary Figure 1: Clinician demographics


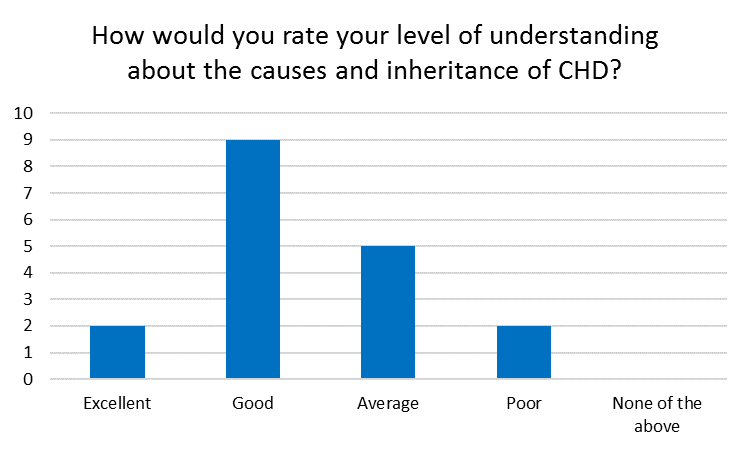

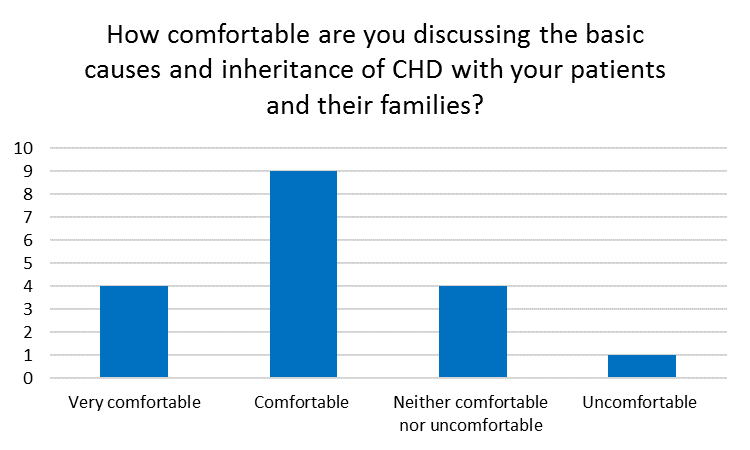

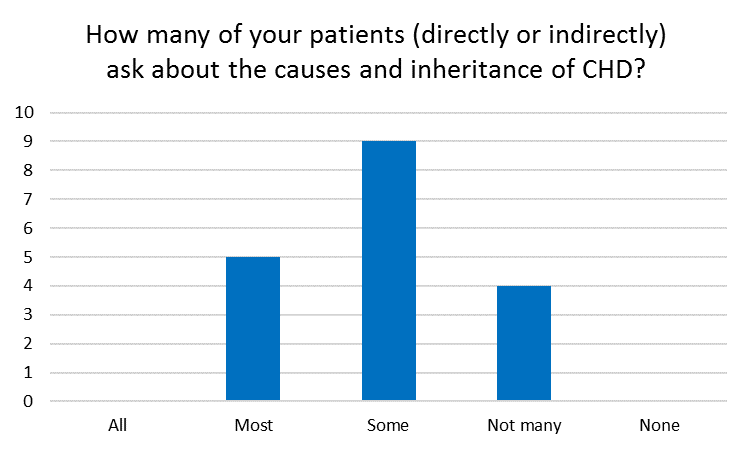

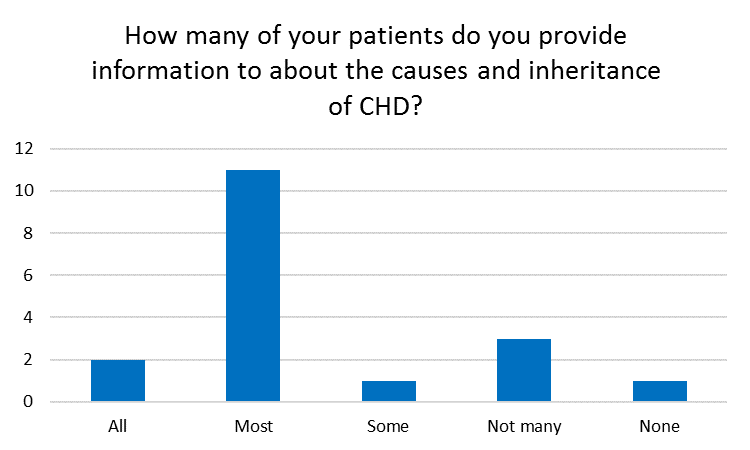

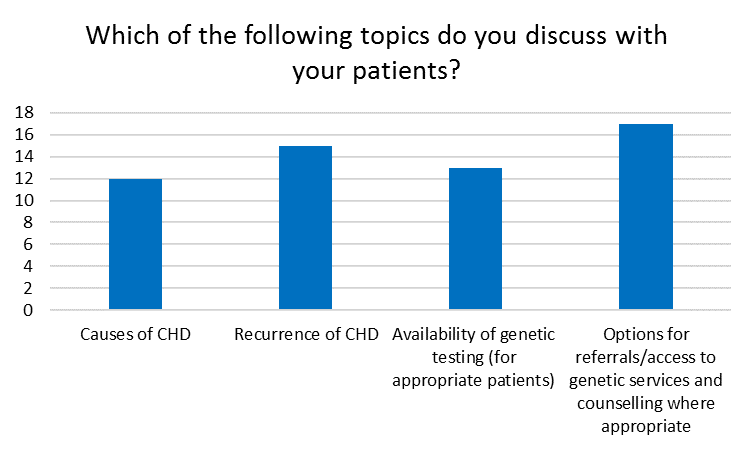

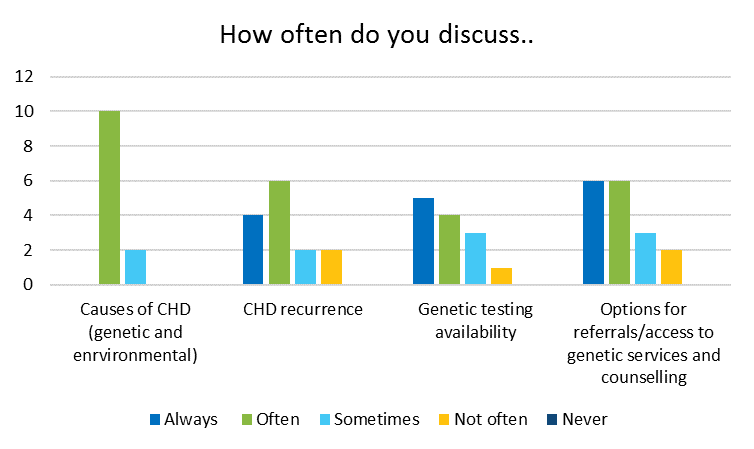

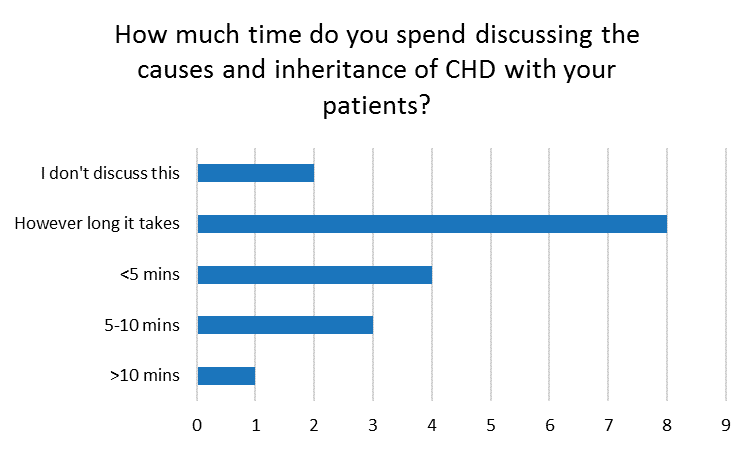


Supplementary Figure 2: Cardiac clinician current practices on the content and delivery of genetics-related information in CHD.
